# Role of Atypical Chemokine Receptors in Microglial Activation and Polarization

**DOI:** 10.3389/fnagi.2017.00148

**Published:** 2017-05-26

**Authors:** Valentina Salvi, Francesca Sozio, Silvano Sozzani, Annalisa Del Prete

**Affiliations:** ^1^Department of Molecular and Translational Medicine, University of BresciaBrescia, Italy; ^2^IRCCS-Humanitas Clinical and Research CenterRozzano-Milano, Italy

**Keywords:** chemokine receptors, neuroinflammation, microglia, atypical chemokine receptors, CCRL2

## Abstract

Inflammatory reactions occurring in the central nervous system (CNS), known as neuroinflammation, are key components of the pathogenic mechanisms underlying several neurological diseases. The chemokine system plays a crucial role in the recruitment and activation of immune and non-immune cells in the brain, as well as in the regulation of microglia phenotype and function. Chemokines belong to a heterogeneous family of chemotactic agonists that signal through the interaction with G protein-coupled receptors (GPCRs). Recently, a small subset of chemokine receptors, now identified as “atypical chemokine receptors” (ACKRs), has been described. These receptors lack classic GPCR signaling and chemotactic activity and are believed to limit inflammation through their ability to scavenge chemokines at the inflammatory sites. Recent studies have highlighted a role for ACKRs in neuroinflammation. However, in the CNS, the role of ACKRs seems to be more complex than the simple control of inflammation. For instance, CXCR7/ACKR3 was shown to control T cell trafficking through the regulation of CXCL12 internalization at CNS endothelial barriers. Furthermore, D6/ACKR2 KO mice were protected in a model of experimental autoimmune encephalomyelitis (EAE). D6/ACKR2 KO showed an abnormal accumulation of dendritic cells at the immunization and a subsequent impairment in T cell priming. Finally, CCRL2, an ACKR-related protein, was shown to play a role in the control of the resolution phase of EAE. Indeed, CCRL2 KO mice showed exacerbated, non-resolving disease with protracted inflammation and increased demyelination. This phenotype was associated with increased microglia and macrophage activation markers and imbalanced M1 vs. M2 polarization. This review will summarize the current knowledge on the role of the ACKRs in neuroinflammation with a particular attention to their role in microglial polarization and function.

## Introduction

The central nervous system (CNS) degeneration, which characterizes several chronic neurodegenerative diseases, such as Alzheimer’s disease (AD), Parkinson’s disease (PD), amyotrophic lateral sclerosis (ALS) and multiple sclerosis (MS), is closely linked to immune activation occurring in the CNS called neuroinflammation (Ransohoff, 2016a). Regardless the triggering mechanisms that can include viral infections, immune-mediate disorders and neuron damage, the immune activation involves microglia and astrocytes (Sofroniew and Vinters, 2010; Perry and Teeling, 2013). These cells represent immune cells resident in the CNS, which regulate CNS homeostasis from the development of brain to the adult life and aging (Schwartz et al., 2013). In particular, any insult is followed by activation of microglia, which culminates with the production of a series of pro-inflammatory mediators that alter brain-blood barrier permeability and induce infiltration of circulating leukocytes inside the CNS (Noh et al., 2014). This effect is regulated by the chemokine system which plays an important role in the control of immune surveillance in the brain (Takeshita and Ransohoff, 2012). The inflammatory response is usually self-limiting and culminates with tissue repair and resolution mechanisms, which mostly involve M2 polarized microglia. However, when this process persists and become chronic, the long-standing activation state of microglia sustains the release of detrimental inflammatory mediators and neurotoxic products which contribute to neurodegenerative sequelae (González et al., 2014; Ransohoff, 2016a).

This review article will focus on the mechanisms involved in the tight regulation of local immune responses occurring during CNS inflammatory process, with particular attention to the role of the chemokine system and atypical chemokine receptors (ACKRs) in microglial activation and polarization.

## Microglial Activation and Polarization

**Figure 1:**
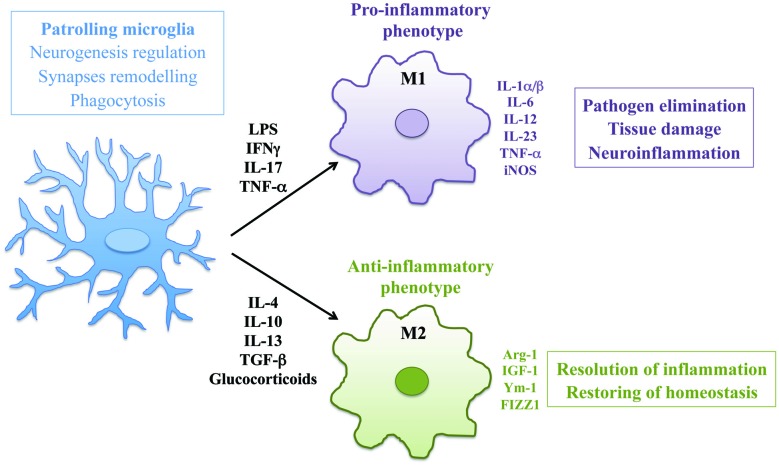
**Activation and polarization of microglia in resting conditions and during neuroinflammation**. The morphology and the phenotype associated with different functional states of microglia are represented. In physiological conditions patrolling microglia regulate central nervous system (CNS) homeostasis. In neuroinflammation microglia assume ameboid morphology and acquire classical M1 or alternative M2 phenotype according to the nature of local milieu.

Microglia are tissue-resident macrophages of the brain and spinal cord, derived from primitive myeloid progenitors in the yolk sac that invade the brain parenchyma during embryonic development (Ginhoux et al., 2010). As mononuclear phagocytes of the CNS, microglia play an important role in maintaining normal brain functions in both physiological and pathological conditions (Wolf et al., 2017). In CNS development, microglia regulate neurogenesis by phagocyting apoptotic newborn cells in the sub-granular zone (Sierra et al., 2010) and support neuronal survival by accumulating around axons and secreting trophic factors like IGF-1 (Ueno et al., 2013). In addition, microglia actively remove the excess of synaptic connections and play a major role in synaptic pruning during postnatal development in mice (Paolicelli et al., 2011). In physiological conditions microglia are responsible to eliminate and remodel synapses and support myelin turnover by phagocytosis of axon terminals and dendritic spines also in adult brain (Tremblay, 2011). Morphology of microglia reveals the different activation states of these cells. In healthy brain, microglia have a ramified shape with extensively branched processes in contact with neurons, astrocytes and blood vessels, that continually survey and monitor changes in the local milieu (Nimmerjahn et al., 2005). Once activated by tissue damage, viral or bacterial insults or pro-inflammatory stimuli these cells develop ameboid morphology characterized by cell body enlargement, shortened cell processes and the presence of numerous cytoplasmic vacuoles, associated with phagocytosis and pro-inflammatory functions (Djukic et al., 2006; Colonna and Butovsky, 2017). Microglia like macrophages are plastic cells able to acquire distinct functional phenotypes according to the nature of the local microenvironment (Figure 1). In addition to morphological differentiation, these cells express different surface markers such as Toll-like receptors, NOD-like receptors, nucleotide-binding oligomerization domain and scavenger receptors. In response to pro-inflammatory stimuli, like LPS, IFNγ and TNF-α, microglial cells produce inflammatory cytokines such as IL-1α/β, IL-6, IL-12, IL-23, TNF-α, iNOS, CCL5 and express Fc receptors CD16, CD32, CD64 and CD68. This pro-inflammatory polarization corresponds to the “classical” M1 activation state (Boche et al., 2013; Cherry et al., 2014). On the other hand, anti-inflammatory stimuli such as IL-4, IL-10, IL-13, TGF-β and glucocorticoids polarize microglia in the “alternative” M2 phenotype that promotes resolution of inflammation and restoring of homeostasis. M2 polarized cells produce anti-inflammatory molecules like arginase-1 (Arg-1), that contributes to matrix deposition and wound healing, growth factors like IGF-1, chitinase 3-like 3 (Ym-1) and FIZZ1 which promote deposition of extracellular matrix and express characteristic receptors of M2 phagocytes such as mannose receptor (CD206) and TREM2 (Raes et al., 2002; Orihuela et al., 2016). Activation of microglial cells strongly influence the pathogenesis of neuroinflammatory and neurodegenerative diseases such as PD, ALS, MS, AD, traumatic brain injuries, stroke and brain tumors (Wolf et al., 2017). However, the concept of microglia polarization is now debated, since M1/M2 paradigm may represent an oversimplification of *in vivo* activation. Transcriptomic and proteomic analysis will help to define the heterogeneity of microglial phenotype associated to different pathological contexts (Ransohoff, 2016b).

## Chemokine System in Neuroinflammation

Chemokines are small, secreted chemotactic cytokines involved in leukocyte trafficking both in homeostatic and inflammatory conditions. Chemokines play their functional role by the engagement of the cognate G protein-coupled receptors (GPCRs), which results in the receptor activation and the triggering of intracellular signaling cascade that regulates several cell activities such as migration, adhesion, phagocytosis, cytokine secretion, proliferation and apoptosis (Bachelerie et al., 2014a). More than 50 different chemokines and about 20 chemokine receptors have been discovered (Bachelerie et al., 2014a). Chemokines are divided into CC, CXC, XC and CX3C subfamilies according to the specific nature of the cysteine motif (Zlotnik and Yoshie, 2000). The chemokine system displays promiscuity features in ligand binding and chemokine receptor expression on different leukocytes (Bachelerie et al., 2014a).

In CNS, chemokines play a role in physiological conditions being involved in neuronal migration, cell proliferation and synaptic activities. During neuroinflammation, in addition to their primary role in leukocytes recruitment, they can also have direct effects on neuronal cells and mediate the cross talk between neurons and inflammatory cells (Cartier et al., 2005). Chemokines can exert neuroprotective role. For instance, CCL2 was described to play a role as a protective agent against the toxic effects of glutamate and HIV-tat-induced apoptosis (Eugenin et al., 2003). In addition, the signaling of the neuronal CX3CL1 and its receptor CX3CR1 were shown to reduce the levels of neurotoxic substances such as TNF-α and nitric oxide in activated microglia during neuroinflammation (Mattison et al., 2013). On the contrary, chemokines can directly induce neuronal death or indirectly through the activation of microglia killing mechanisms (Sui et al., 2006; Yang et al., 2011). A new role for chemokines in the modulation of the release of neuropeptides and neurotransmitters was also proposed (Rostène et al., 2007, 2011), as shown by the effect of CXCL12 in tuning the firing pattern of vasopressin neurons (Callewaere et al., 2006).

In active MS patients, the pathogenic Th1 axis, involving CXCL10, CCL3, CCL4, CCL5 and the receptors CXCR3 and CCR5, is activated in blood and cerebrospinal fluid (Uzawa et al., 2010). On the contrary, the Th2 receptors CCR4 and CCR3 and the ligand CCL2 play a protective role (Nakajima et al., 2004). Th17 are recruited by CCR6/CCL20 axis expressed by epithelial cells of choroid plexus as shown in the experimental autoimmune encephalomyelitis (EAE) model (Reboldi et al., 2009; Cao et al., 2015). CXCL12 acts as double edge sword since it is responsible for the recruitment of monocytes in the CNS during MS (Azin et al., 2012) and, at the same time, contributes to remyelination and neuroprotection processes (Khorramdelazad et al., 2016). Moreover, in MS, CXCL13 secreted by microglia has been shown to be involved in the recruitment of CXCR5 expressing Th1, Th17 and B cells (Huber and Irani, 2015).

The dysregulation of the CX3CL1-CX3CR1 communications between neurons and microglia has been investigated in AD patients showing opposite effects. In one study CX3CR1 deficiency was reported to reduce beta-amyloid deposition in AD experimental model (Lee et al., 2010). Other work showed different contribution of the soluble vs. the membrane-bound forms of CX3CL1 in the regulation of microglial phagocytosis of plaques (Cho et al., 2011; Lee et al., 2014). CX3CL1 gene expression was upregulated in the brain of AD patient (Strobel et al., 2015). Other chemokines, such as CCL3, CXCL10 and CCL2 were implicated in the regulation of glial activity involved in neuroinflammation and neurotoxic environment during the AD development (Xia et al., 2000; Galimberti et al., 2006; Meyer-Luehmann et al., 2008; Dos Passos et al., 2016). A relevant role of glial activation in the development of neuroinflammation associated with damage of the nigro-striatal dopaminergic system was suggested in PD (Przedborski, 2010). The CXCL12 and CXCR4 expression is increased and can promote apoptosis of dopamine neurons (Shimoji et al., 2009). On the contrary, CX3CL1 exerts a neuroprotective effect during PD development (Pabon et al., 2011).

Overall, neuroinflammation is a complex process involved in several CNS diseases ranging from autoimmune to degenerative pathologies and chemokine system plays key role in immune surveillance, in microglial activation and functions, in neurocommunications among several CNS cell types and can orchestrate both neuroprotective and detrimental effects.

## Atypical Chemokine Receptors in Neuroinflammation

ACKRs represent a small subset of proteins expressing high degree of homology with chemokine receptors. Since ACKRs lack structural domains required for proper Gα_i_ signaling, they are unable to activate canonical G protein-dependent receptor signaling and chemotactic functions (Bachelerie et al., 2014a,b). At the moment, the ACKR family includes four proteins, namely ACKR1, ACKR2, ACKR3 and ACKR4. Other two proteins, namely CCRL2 and PITPNM3 have been provisionally included with the name of “Ackr5” and “Ackr6”, respectively, but they still need formal approval (Bachelerie et al., 2014b). In the last few years, the role of ACKRs is gradually clarifying since they were shown to regulate inflammation acting as scavenger receptors, promoting chemokine transcytosis or regulating chemokine gradient formation (Mantovani et al., 2001; Nibbs and Graham, 2013; Bachelerie et al., 2014a,b; Bonecchi and Graham, 2016). The role of ACKRs in neuroinflammation is described below and summarized in Table 1.

**Table 1:** **Atypical chemokine receptors (ACKRs) and their role in neuroinflammation**.

Receptor name	Alternative names	Sites of expression	Role in neuroinflammation	References
**ACKR1**	Duffy antigen receptor for chemokines (DARC), CD234	Erythrocytes Vascular endothelial cells Purkinje cells	On erythrocyte it acts as chemokine reservoir On endothelium it contributes to EAE pathogenesis	Minten et al. (2014)
**ACKR2**	CCBP2, D6, CMKBR9	Lymphatic endothelial cells Innate-like B cells Keratinocytes Trophoblasts	It is required for generating T cell responses in EAE It suppresses Th17 responses in EAE	Liu et al. (2006), Hansell et al. (2015)
**ACKR3**	RDC1, CXCR7, CMKOR1	Endothelial cells Hematopoietic cells, Mesenchymal cells Neurons Astrocytes	It contributes to EAE pathogenesis It mediates chemotaxis of activated microglia during EAE	Cruz-Orengo et al. (2011b); Bao et al. (2016)
**ACKR4**	CCRL1, CCXCKR, CCR11	Thymic epithelial cells Keratinocytes Lymphatic endothelial cells	It delays the onset of EAE and reduces disease severity	Comerford et al. (2010)

### ACKR1

ACKR1 binds, with high affinity over 20 inflammatory chemokines belonging to the CC and CXC families (Novitzky-Basso and Rot, 2012). ACKR1 is expressed on erythrocytes, cerebellar Purkinje cells, postcapillary venules and capillary endothelial cells. Depending on its cellular expression, the biological functions of ACKR1 are quite different. Indeed, ACKR1 expressed by erythrocytes regulates the bioavailability of circulating chemokines by binding them with high affinity (Schnabel et al., 2010), while endothelial ACKR1 induces chemokine internalization and transcytosis from the basolateral to the luminal side of endothelium, where the chemokines are immobilized and contribute to leukocyte extravasation *in vivo* (Pruenster et al., 2009). In neuroinflammation, ACKR1 is upregulated on endothelial cells in CNS microvessels during EAE and in MS where it mediates the abluminal to luminal transport of inflammatory chemokines across the blood-brain barrier and contributes to EAE pathogenesis (Minten et al., 2014). In bone marrow chimera experiments, ACKR1 expression in erythrocytes was shown to be responsible for the increased plasma levels of inflammatory chemokines observed during EAE. However, for the full development of the disease, the expression of ACKR1 was necessary also on endothelial cells (Minten et al., 2014).

### ACKR2

ACKR2 binds a broad panel of inflammatory CC chemokines and is expressed by lymphatic endothelial cells, trophoblasts, keratinocytes and some leukocyte subsets, mostly innate-like B cells (Lee et al., 2013). ACKR2 binds and internalizes its ligands and target them for lysosomal degradation. Therefore, ACKR2 is a chemokine-scavenging receptor, aiding the resolution of inflammatory reactions (Graham, 2009). Studies in EAE model suggested that ACKR2 functions may be context-dependent. Indeed, ACKR2 KO mice are unexpectedly resistant to the induction of EAE due to impaired encephalitogenic responses (Liu et al., 2006). This observation is ascribed to defective T-cell priming resulting by the suppression of dendritic cells migration to the lymph nodes caused by excessive inflammation at the immunization site (Liu et al., 2006). In contrast, ACKR2 deficiency was recently associated with an exacerbated EAE phenotype when mice were immunized with the entire protein but not the MOG peptide, suggesting a regulatory role for ACKR2 mediated by T cell polarization (Hansell et al., 2015).

### ACKR3

ACKR3 binds two chemokines, namely CXCL12 and CXCL11. ACKR3 is expressed by endothelial cells, some hematopoietic cells, mesenchymal cells, neurons and astrocytes. ACKR3 is able to internalize CXCL12 and modulate CXCR4 expression and signaling by forming heterodimers with CXCR4 (Levoye et al., 2009; Décaillot et al., 2011). Thus, ACKR3 can control chemokine responsiveness by regulating CXCR4 protein levels and CXCL12 scavenging activity as shown in migrating cortical interneurons (Sánchez-Alcañiz et al., 2011; Abe et al., 2014). Loss of CXCL12, a chemokine that restricts the CNS entry of CXCR4-expressing leukocytes from abluminal surfaces of blood-brain barriers, has been described in MS. During EAE, ACKR3 expression on endothelial barriers is increased at sites of inflammatory infiltration (Cruz-Orengo et al., 2011b) and its activation, by scavenging CXCL12, is essential for leukocyte entry via endothelial barriers into the CNS parenchyma (Cruz-Orengo et al., 2011b). Administration of ACKR3 specific antagonist CCX771 increases abluminal levels of CXCL12 at the blood-brain barrier, preventing the pathological entry of immune cells into CNS parenchyma, thus improving clinical signs of EAE disease (Cruz-Orengo et al., 2011b). In addition, ACKR3 antagonism during EAE helps preserving axonal integrity (Cruz-Orengo et al., 2011a). In mice with EAE, ACKR3 is also upregulated in migrating oligodendrocytes progenitors, important cells for the remyelination process, in the subventricular zone (Banisadr et al., 2016). More recently, it was reported that ACKR3 expression is responsible for the migration of activated microglia and positively correlated with the clinical severity of EAE (Bao et al., 2016). Indeed, an ACKR3 neutralizing antibody modulates microglial chemotaxis and ameliorates EAE symptoms (Bao et al., 2016).

### ACKR4

ACKR4 binds the homeostatic CC chemokines CCL19, CCL21, CCL25 and with lower affinity CXCL13. ACKR4 is expressed by thymic epithelial cells, keratinocytes and lymphatic endothelial cells. After chemokine binding ACKR4 internalizes and drives its ligand to degradation (Comerford et al., 2006), thus representing the homeostatic counterpart of the inflammatory CC chemokine scavenger receptor ACKR2. In an EAE model, ACKR4 deficient mice developed the disease earlier and with more severity compared to wild type mice. This earlier onset was associated with an enhanced splenic Th17-type response, an elevated expression of IL-23 transcript, and an increase in CCL21 in the CNS (Comerford et al., 2010).

## Role of CCRL2 in Microglial Polarization

CCRL2 is a seven transmembrane protein that shares some structural and functional aspects with ACKRs, such as the lack of conventional GPCR signaling and the inability to induce cell migration (Zabel et al., 2008; Del Prete et al., 2013; Bachelerie et al., 2014a; De Henau et al., 2016). Some authors described binding and/or functional activation of CCRL2 in response to chemokines CCL2, CCL5, CCL7, CCL8 and CCL19 (Biber et al., 2003; Catusse et al., 2010; Leick et al., 2010). However, these results were not subsequently confirmed by other groups (Del Prete et al., 2013; De Henau et al., 2016). CCRL2 binds and presents chemerin, a non-chemokine chemotactic protein, to adjacent cells expressing the functional chemerin receptor ChemR23, and acts as regulator of chemerin bioavailability (Zabel et al., 2008; Gonzalvo-Feo et al., 2014). Neither internalization nor calcium mobilization was described upon chemerin binding (Zabel et al., 2008). CCRL2 is expressed by endothelial and epithelial cells, and by a variety of leukocytes, including macrophages, dendritic cells, mast cells and neutrophils and its expression is increased by pro-inflammatory stimuli (Galligan et al., 2004; Zabel et al., 2008; Otero et al., 2010; Monnier et al., 2012; Del Prete et al., 2013; Gonzalvo-Feo et al., 2014). In particular, CCRL2 was described to play a non-redundant role in the regulation of dendritic cell migration during airway inflammation (Otero et al., 2010). Moreover, *in vitro* and *in vivo* studies showed CCRL2 expression in mouse microglia and astrocytes and upregulation in macrophages in the EAE model (Zuurman et al., 2003; Brouwer et al., 2004). To better understand the role of CCRL2 in CNS physiopathology, our group studied the role of CCRL2 deficiency in EAE model (Mazzon et al., 2016).

CCRL2 expression was found upregulated following MOG_aa35–55_ immunization and paralleled the kinetics of the clinical symptoms, reaching the peak induction at the acme of the disease and declining thereafter (Mazzon et al., 2016). During the development of EAE, the upregulation of CCRL2 was associated with increased levels of chemerin (Graham et al., 2009; Mazzon et al., 2016), mimicking the regulation of ACKR3 and its ligand CXCL12 (Banisadr et al., 2016). CCRL2 deficient mice showed exacerbated EAE clinical phenotype, in terms of increased mortality, higher maximum and cumulative clinical score. The histopathological analysis revealed an intense and persistent inflammatory reaction associated with increased demyelination in CCRL2 deficient spinal cords at the recovery phase of the disease. These observations were paralleled by the increase in T cell infiltration in CCRL2 deficient mice. In addition, in CCRL2 KO mice, the macrophage/microglia activation markers, namely Iba-1, CD68, and TREM2 remained elevated during the recovery phase of the disease. An unbalanced M1/M2 polarization, with a persistent predominance of the M1 markers was also observed during the recovery phase of the disease. This was in contrast to control mice that developed all the signs of resolution of inflammation including the M2 phenotype characterizing the CNS regenerative response. These results candidate CCRL2 as an important player in EAE-associated inflammatory reactions and suggest the tuning of microglial activation and polarization as an additional mechanism used by ACKRs to regulate CNS inflammatory responses.

## Conclusions

Neuroinflammation is a key component of neurodegenerative diseases. This process involves the effector components of both innate and adaptive immunity. In the CNS, microglia represent the resident immune component involved in tissue homeostasis. Being an immune sensor of altered homeostasis, microglia accumulate in response to neuron injury and after the entry of foreign substances in the CNS. The way microglia become activated represents a crucial element in the regulation of neuroinflammation and may be associated with either beneficial or detrimental effects resulting in neuroprotection or neurotoxicity. The chemokine system plays multiple roles in the modulation of this multifaceted process. Our growing understanding on the functions of the ACKRs increases the level of complexity of this scenario and provides new potential targets to be exploited in the control of neuroinflammation.

## Author Contributions

VS and FS wrote and edited the manuscript and prepared the Table and Figure. SS and ADP contributed in writing and supervised the final version of the manuscript.

## Conflict of Interest Statement

The authors declare that the research was conducted in the absence of any commercial or financial relationships that could be construed as a potential conflict of interest.
